# Reactive Agility and Pitching Performance Improvement in Visually Impaired Competitive Italian Baseball Players: An Innovative Training and Evaluation Proposal

**DOI:** 10.3390/ijerph20126166

**Published:** 2023-06-18

**Authors:** Giuditta Carretti, Raffaele Bianco, Eleonora Sgambati, Mirko Manetti, Mirca Marini

**Affiliations:** 1 Section of Anatomy and Histology, Department of Experimental and Clinical Medicine, University of Florence, 50134 Florence, Italy; 2 Department of Biosciences and Territory, University of Molise, 86090 Pesche, Italy

**Keywords:** visual disability, athletic performance, blind baseball, pitching performance, adapted training, adapted sport

## Abstract

Visual input significantly affects kinesthesis skills and, hence, visually impaired individuals show less developed sensorimotor control, especially in an unfamiliar outdoor environment. Regular blind baseball practice can counteract such a deficit but, given the complex kinetic chain model required, a targeted workout proposal is needed to improve the main athletic gesture performance. On these premises, we investigated, for the first time, the running and pitching performance of a competitive Italian blind baseball team through quantitative tools and parameters such as Libra Easytech sensorized proprioceptive board, goniometric active range of motion, chronometric speed, and pitching linear length. Moreover, the perceived physical exertion was assessed by the Borg CR10 scale. Consequently, an adapted athletic training protocol was designed and tested on the field during the competitive season, with the aim to strengthen sport specific-gesture coordination and efficacy as well as to prevent injuries. Quantitative assessments showed an improvement in ankle stability index, bilateral upper limb and hip mobility, reactive agility, running braking phase control during second base approaching, and auditory target-related pitching accuracy along with a decrease in perceived physical exertion. This protocol might therefore represent an effective and easily reproducible training and evaluation approach to tailor management of visually impaired baseball players, and safely improve their athletic performance under the supervision of an adapted exercise specialist.

## 1. Introduction

According to the World Health Organization data, across the world, 188.5 million people suffer from mild vision loss, 217 million from moderate to severe loss, and 36 million are blind [1]. Indeed, visual disability is one of the most frequent functional impairments and, given the sight pivotal role in human interaction with the surrounding environment, it heavily affects psychophysical and social functioning [2,3]. Notably, it has been widely reported that visually impaired subjects tend to have less developed motor and orientation skills, especially in static and dynamic balance, postural control, proprioception, and coordination [4,5,6]. Visual deprivation implies negative alterations in gait parameters, global and segmental body kinematics, bilateral and head–trunk coordination, and core stability, heavily challenging the mobility of blind people, particularly in an unfamiliar outdoor environment [7,8]. Head–trunk coordination, which is motor development-dependent, is a crucial requisite for functional locomotion and goal-oriented movements on the horizontal plane. Sensory disabilities delay the whole development process, causing, specifically, sensorimotor control deficits in segmental coordination and thus affecting spatial performance efficacy and accuracy [9,10,11]. In particular, it has been shown that straight-ahead auditory targets facilitate localization, while precision is higher in the central rather than lateral direction in visually impaired subjects [12,13]. This may depend on the specific difficulty separating head and trunk movements, especially during rotation around the vertical axis, as well as disability-related upper body tension and stiffness [14,15]. As a result, these individuals often deal with movement limitations both in daily life and leisure/sport activities and, if this motor experience lack is not intentionally compensated, it can easily lead to sedentary life-related physical and mental disorders [16,17]. Spatial awareness and management are generally accomplished via integration of visual, vestibular, and proprioceptive cues [18,19,20], but in case of visual impairment, subjects strongly rely on egocentric references and acoustic feedback [21,22,23]. Although visual inputs significantly contribute to orienteering functions and, hence, affect kinesthesis abilities and athletic performance, regular adapted sensorimotor training based on vicarious senses can improve such skills in visually impaired subjects [6,24]. When vestibular and proprioceptive control are systematically stimulated through structured workout proposals aimed at their mastery, visually impaired individuals are capable of properly performing not only daily functional tasks, but also complex athletic ones [25,26,27].

Since physical exercise benefits for disabled people have long been known [28,29,30], the participation of visually impaired athletes in sport has been allowed and regulated starting from the beginning of the last century in the United States [31]. At first, only the most popular disciplines such as athletics, gymnastics, and baseball provided a blind adapted version while, nowadays, this target population can compete in several sports, in single or combined official classes [32,33]. In such a context, Italian blind baseball (BXC), inspired by the well-known American game but specifically designed for sight-impaired players, is a team sport practiced worldwide. BXC includes players with a mix of visual disability degree, age, and gender, as described in detail in our previous studies [27,34]. Given its peculiarities and the kinetic chain model required to effectively perform the three main basic skills (i.e., running, batting, and pitching), this team sport represents not only a collective outdoor activity but also a valid opportunity to strengthen the confidence, safety, and coordination of visually impaired subjects while purposefully moving within an unfamiliar environment [35]. Despite the increasing popularity of this discipline and the well-acknowledged high performance level and perceptual, cognitive, and motor skills needed to manage the uncountable ever-changing gaming situations required by players in official competitions, little is known about the specific movement patterns and related injury risk [36,37]. Therefore, in the present study we investigated, for the first time, the running and pitching performance parameters of the whole Fiorentina BXC blind baseball team of Florence with the goal of designing a novel, specific, evaluative, and training approach to maximize performance and prevent injuries during the competitive season.

## 2. Materials and Methods

### 2.1. Participants

The whole Fiorentina BXC team, which competes at the national level and is registered in Polisportiva Silvano Dani, provided signed informed consent and agreed to participate in the training and testing activities promoted by the management during the whole professional championship season. Concerning any possible physical risk, all athletes were in possession of a valid sport medical certificate issued by a sports doctor as mandatorily required by the Official Federation (FIBS) to take part to the regular competitive championship. In such a perspective, the training and evaluation protocol was conceived, supervised, and performed by the official technical staff of the team after receiving the president’s approval. Since the sample consisted of a professional sport team whose athletes are regularly trained and measured at various stages of their competitive season, no formal approval by a properly constituted ethics committee was applicable.

Specifically, the investigated group consisted of eight visually impaired athletes, mixed in age, gender, and visual disability degree. All study procedures were conducted following the rules of the Declaration of Helsinki of 1975 [38], revised in 2013. For the purposes of this study, the data were treated and processed in a completely anonymous form.

### 2.2. Participant Evaluations

**Figure 1:**
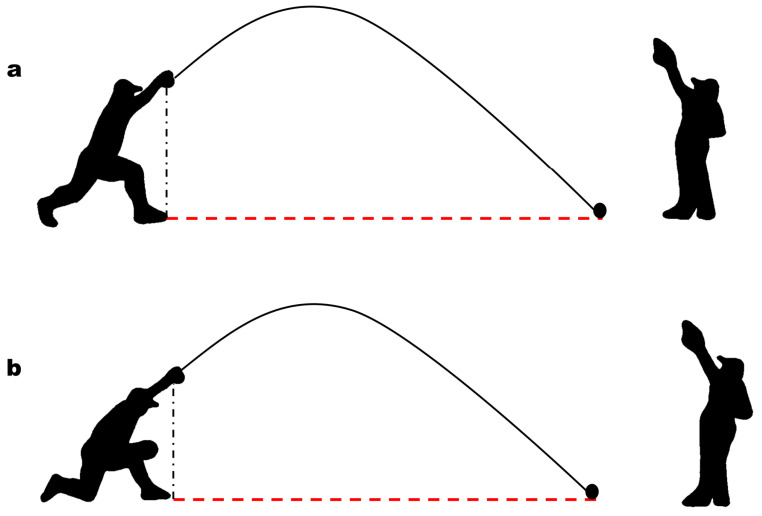
Pitching linear length assessment procedure. Investigated athletic gesture performed by visually impaired pitcher (left black silhouette) both in orthostatic (**a**) and kneeling (**b**) position, placing the dominant upper limb overhead and following the auditory target provided by the sighted catcher (right black silhouette). The ball has a parabolic trajectory (black curve line) from hand release point to the first landing mark. The horizontal distance between ball release point orthogonal projection on front foot and the landing mark can be equated with pitching linear length (red dotted line) and assessed using a track and field measuring tape.

Baseball player evaluations were conducted during the in-season phase (i.e., from May to July as mandatorily scheduled by the Official Federation). At baseline and after ending the structured adapted athletic training (AAT) intervention, all participants underwent quantitative evaluation and test battery including limb goniometric active range of motion (ROM), chronometric running speed, and ball pitching linear length measurement. Concerning the assessment of mobility, the main upper and lower limb joints were considered, namely those of the shoulder, elbow, and wrist, as well as the hip and knee. The ROM of all these joints was standardly measured using a manual goniometer and placing the subject in orthostatic position, thus recalling the real postural alignment and load experienced on game field. Specifically, ROM measurement was performed taking the anatomical site as “0” regardless of the investigated movement. To evaluate running speed, each visually impaired athlete was asked to race from home plate to second base by turning around the first base guided by the different sound inputs that are provided in the official regulation [39]. Given the articulated running pathway and the collision risk by placing dual-beamed photocells on or in proximity to it, running time was safely clocked through a manual chronometer at the first-base transition and at the second base arrival; each runner performed two consecutive attempts. Such a running task represents the most intense physical exertion during regular matches and, therefore, a verbal version of the Borg CR10 scale was administered right after it to appreciate the physical effort experienced [40]. Then, since the official field measures were known, speed was calculated applying the physics formula (i.e., distance/time) using the best time achieved. Pitching linear length was evaluated by placing each subject in their playing position according to the role held in the defending game phase. The players were instructed to perform the athletic gesture, both in orthostatic and kneeling position, placing the dominant arm overhead and trying to reach the target auditory provided by the sighted catcher on the second defending base. Two attempts were given for each position, and the higher length was registered. As far as the measurement procedure was concerned, given that ball describes a parabolic trajectory from overhead release point (orthogonally projectable on front foot) to first landing point, the horizontal distance between these two references can be equated with pitching linear length and consequently assessed using a track and field measuring tape (Figure 1). Moreover, in order to obtain an accurate ball landing mark, red clay court was properly smoothed by the investigator between the two attempts required.

In addition, given the competitive season injury prevention goal of our intervention, ankle stability was dynamically evaluated through the validated Cauquil-De Gunsch (CDG) test provided by the Libra Easytech sensorized proprioceptive board software (Easytech, Borgo San Lorenzo, Florence, Italy) [41]. This device consists of a 42 × 42 cm anti-slip surface-coated square unstable platform that allows only one degree of freedom at a time, in the frontal or sagittal plane, which is connected via USB to a computer. Three tilting radius wedges 5, 12, or 20 cm in size can be used to adjust the exercise difficulty level. The subject, who can be tested in various positions with a maximum measurement error to 0.2 degrees, is required to keep the board in balance, parallel to the floor. The software provides four different course patterns: straight line, sinusoid, square, or triangular wave. The difficulty degree range is displayed on the screen as two parallel lines placed on each side of the track and each time these lines are approached or crossed, a peculiar acoustic feedback is played. Specifically, during the CDG test, the investigator examines the subject in bipodalic, and left and right monopodalic stances strictly following the preset parameters recommended by the manufacturer. At the end of trial, the recovery ratio index, expressed as percentage, can be considered a significant ankle stability predictor. According to the cut-off provided by the instruction manual [42], lower values correspond to a lower ankle sprain risk. In addition, the athletes were invited to fill out an online survey questionnaire created using the Google Forms platform and sent as direct link by e-mail [6,34]. The responses were completely anonymous and confidential. The baseline survey consisted of sociodemographic characteristics including age, gender, and information on visual disability type and severity (i.e., total blindness, severely sight-impaired, and mildly sight-impaired according to the Classification of the International Blind Sports Federation) [33]. In addition, a post-AAT survey investigated the specific motor protocol experience satisfaction, as well as the adapted physical exercise specialist’s competency. Study participants were also requested to indicate if they would continue performing AAT as a part of their training routine and any perceived benefits in their athletic performance. Finally, subjects were asked to report if they experienced the protocol as a strenuous physical activity, and if it corresponded to their expectations.

### 2.3. Adapted Athletic Training Protocol

**Figure 2:**
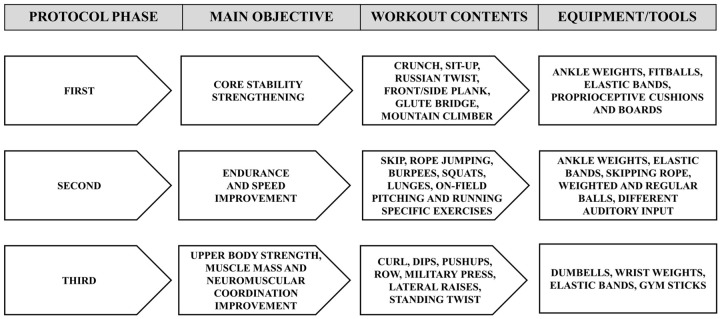
Flowchart of the adapted athletic training protocol.

The tailored workout protocol aimed to follow competitive season pace without causing any psychophysical overload, and was conceived as an 8-week intervention, from May to July 2022, performed on field as a single 90 min session every week. Considering the needs and safety of visually impaired athletes and the team heterogeneity, a circuit training methodological approach was applied. This methodology was chosen in order to respect the individual fitness and residual skill level and simultaneously facilitate exercise explanation/demonstration, as well as autonomous subject spatio-temporal orientation. Since reactive agility, running speed, and pitching power and precision—training protocol main objectives—are multifactorial parameters, both general and specific exercises were performed to strengthen the anatomo-functional prerequisites, global/segmental coordination, and sport specific-gesture kinetic chain activation and control. Finally, given the progressive load increase typical of the championship season that is regularly played during a particularly hot period such as the Italian summer, the intervention was organized in three phases characterized by a gradually enhanced psychophysical effort. In detail, since the trunk plays a key role in motor control, balance, and force delivery from the lower to upper limbs, the first phase focused on core stability improvement. The second phase aimed to enhance physical endurance and running speed through simulated competitive game frames. Lastly, the third phase was centered on increasing upper body strength, muscle mass, and neuromuscular coordination, with the aim of boosting technical gestures power while reducing shoulder and elbow injury risk. Each workout session was designed and led by recalling the pre-game and in-game situations, thus fastening the adaptability, and concurrently promoting the competitive mindset and protocol adherence of the athletes. The specific objectives, contents, and equipment of each phase of the AAT protocol are detailed in Figure 2. As examples, two detailed workout schedules focusing on the running and pitching training are available as Supplementary Materials.

### 2.4. Statistical Analysis

All data are represented as mean ± standard error of the mean (SEM), mean ± standard deviation (SD), or percentage of subjects. The Wilcoxon signed-rank test was used to compare the baseline vs. post-AAT scores. Values of *p* < 0.05 were considered statistically significant. Statistical analyses were performed using the SPSS version 28.0 (Statistical Package for the Social Sciences, Chicago, IL, USA).

## 3. Results

Eight visually impaired baseball athletes (62.5% male; mean ± SD age, 25.4 ± 9.1 years) took part in this study. The sociodemographic characteristics and visual disability features of the study participants are detailed in Table 1. In particular, six subjects had congenital blindness, while the other two had acquired blindness. As far as the two subjects with acquired vision loss are concerned, one declared that she was blind for over 10 years while the other one that he was blind for 3 to 5 years.

Table 2 displays data concerning the assessment of goniometric active ROM of different anatomical joints at baseline and after ending the structured AAT protocol. The overall ROM values showed a trend toward an improvement after the tailored protocol (Table 2). Of note, a significant increase in the mean values of bilateral wrist flexion and hip flexion–extension along with bilateral shoulder abduction was observed after the AAT intervention compared to baseline (Table 2).

As reported in Table 3, no significant difference in running speed could be detected between baseline and post-AAT. However, a trend toward a decrease in running speed from home plate to second base was found post-AAT with respect to baseline (Table 3). Instead, running speed from home plate to first base tended to be higher post-AAT compared with the baseline (Table 3).

Table 3 also displays the mean scores of overhead pitching length performed in both orthostatic and kneeling positions. The higher, though not statistically significant, post-AAT protocol scores revealed a trend toward an improvement (Table 3). In addition, Table 3 shows the results obtained by the CDG test performed on Libra Easytech proprioceptive balance board. Notably, all the evaluated parameters (i.e., bipodalic and monopodalic stance), which are strongly correlated with ankle stability index, demonstrated a statistically significant improvement (Table 3). A significant decrease in perceived physical exertion, assessed by an adapted verbal version of the Borg CR10 scale, was also observed post-AAT (Table 3).

As far as the assessment of the protocol experience satisfaction is concerned, the post-AAT data showed that 100% of the enrolled athletes were satisfied with the protocol practice and the competency of the adapted physical exercise specialist. Although 50% of participants considered the intervention as strenuous, 87.5% of them reported that they would willingly continue performing it as part of their training routine. The same percentage of subjects also reported that the practice of this specific AAT protocol provided benefits in their athletic performance. Finally, this experience satisfied the expectations of the whole athlete sample.

## 4. Discussion

It has been widely demonstrated that visual disability causes adaptation strategies oriented to more cautious motor patterns, especially in a noisy outdoor environment [36]. Visually impaired athletes often experience fear of falling during sport practice and, therefore, they run approximately 30% slower, keep longer foot–ground contact, and show a shorter stride length and a reduced hip ROM when compared to sighted peers [43]. Moreover, since hearing is confined to the head level and plays a role in postural adjustments in cases of vision loss, such athletes tend to lean their trunk forward to maximize auditory input from their feet during running [44], which negatively affects balance and speed [45]. Therefore, athletic trainers managing visually impaired competitive players should always carefully consider not only their anatomo-functional characteristics, but also their disability-related compensatory strategies to increase sport-specific performance and prevent injuries [46]. On this basis, the ankle stability and postural control were investigated using a quantitative tool such as Libra Easytech that allowed us to simultaneously obtain anatomic, functional, and sensorimotor parameter-related outcomes. Moreover, in the present study, the CDG test was conducted on athletes wearing the mandatory blindfold mask, reflecting their residual and adaptive skills in game conditions. The statistically significant results concerning ankle stability index, along with no occurrence of lower limb injuries during the competitive season, indicates that targeted sport-specific proprioceptive exercises should be incorporated in blind baseball athletic training. In agreement with the aforementioned findings, it has been previously reported that stabilization and balance exercises performed on unstable supports significantly improve kinesthetic analyzer development leading to an increase in reactivity, neuromuscular coordination, and functional joint work [46]. There is also evidence to indicate that weak ankle flexor muscles or limited loaded dorsiflexion deeply affect dynamic balance, and hence, represents the major leading cause of falls and ankle sprain [26]. Indeed, improving ankle stability through a sport-specific sensorimotor training in dynamic frames could positively influence balance and proprioceptive postural control, both anticipatory and reactive, thus contributing to injury prevention especially in sight-impaired individuals [47,48]. In addition, lower limb proprioceptive feedback, particularly enhanced by performing simulated jogging movements on unstable tools, can help eliminate feet–ground contact delays and unnecessary muscular/joint stress during running [49,50,51].

Especially considering the shortness of the present intervention, the post-AAT increase in bilateral hip ROM and improvement in home plate–first base running speed suggest that training structural and functional requisites through either a segmental or total body approach can accelerate sensorimotor learning, consciousness, and adaptation of visually impaired players. According to the BXC regulation, the batter-runner must touch the offensive second base cushion without committing offensive interference and stopping without overcoming the safety zone [39]. In order to do so, players exclusively follow the auditory input provided by the sighted clapper placed on second base. Therefore, the decrease in first base–second base running speed obtained post-protocol, as well as the lack of interference penalties committed by athletes during the competitive season, might suggest an upgraded braking phase management and an enhanced auditory input-related sensorimotor control. Body segments must be sequentially activated along the kinetic chain to functionally perform athletic tasks [52,53]. A safe and effective pitching kinetic chain involves highly refined and coordinated segmental movements throughout the body [54]. In addition, more than 50% of the kinetic energy and force during this complex motion is produced by the trunk. Throwing a ball, especially with an overhead-positioned arm, places high stress on several musculoskeletal structures such as the ankle, spine, shoulder, elbow, and core [55]. Owing to trunk–pelvis and head–trunk coordination deficits, visually impaired athletes are not spontaneously able to effectively recruit and activate core muscles during pitching, thus requiring specific training [56]. Targeted exercises combining core stability and trunk/hip mobility are therefore a valid tool both in a performative and preventive perspective [3]. Concerning the pitch gesture in blind baseball, it must be specified that, in contrast with the traditional game, only position pitchers are used as the ball is not thrown toward an opponent batter, but a sighted teammate catcher [39]. Despite this safety aimed adaptation and the consequent lower ball velocity requested, this motor gesture can often result in upper limb injuries [57]. Hence, this investigation focused, for the first time in blind baseball, on pitching length and accuracy instead of ball velocity [58]. Through the proposed exercises aimed to promote upper limb mobility, strength, and proprioceptive control, an improvement in both aforesaid parameters was achieved at the post-intervention evaluation. Finally, despite the workout protocol being short and performed during the competitive season in hot weather conditions, it is noteworthy that the whole team reported a significant improvement in physical exertion perception as testified by a decrease in the Borg CR10 scale score post-AAT.

Since there is a consistent lack of literature dealing with quantitative analysis of performance parameters in visually impaired athletes, this study may represent an effective and easily reproducible evaluative approach to apply on larger scale. Based on objective outcomes, the described experience testifies that it is possible to design, lead, and monitor a targeted workout aimed at improving sport-specific performance without any psychophysical overload while respectful of disability-related residual skills. Finally, our findings highlight that the integration of an adapted physical exercise specialist in the sport team staff should be highly recommended to tailor the management of this target population with a safe and effective improvement of the athletic performance.

Considering that the official BXC regulation allows and encourages mixed teams in terms of age, gender, disability type, and ethnicity, the comparison of two different teams, recruited as experimental and control groups, would not have been correct due to their heterogeneous features. Similarly, the comparison of sedentary visually impaired subjects with athletic peers could have only highlighted the well-known evidence of exercise benefits in this target population [6,26,27,28,29]. Therefore, the main limits of the present study may relate to the small size and the aforementioned heterogeneity of the sample which certainly involved multidimensional parameters and genetic factors which deserve further in-depth investigations in a multidisciplinary perspective.

## 5. Conclusions

In conclusion, the present work could help spreading research interest/application in such an under-investigated field thus promoting future studies involving larger samples of players and multidisciplinary teams. Though trying to overcome the team heterogeneity-related limits of this adapted sport, it is highly recommended to prioritize such a peculiarity in order to properly design further studies and protocols without losing sight of the real game frame. At the same time, BXC teams should be encouraged to progressively integrate adapted exercise protocols, led by graduated specialists, during the entire sport season. Hence, the presented in-season protocol could facilitate such implementation, not necessarily adding training sessions but integrating them in the regular team schedule, thus respecting the psychophysical load and agenda of the athletes. Lastly, given its on-field approach, this pioneering investigation could provide a methodological hint that is easily reproducible and adjustable to the specific features and needs of each BXC team, thus fostering and sharing topic-specific knowledge.

## Figures and Tables

**Table 1:** Demographic data and visual disability features of baseball athletes.

Variables	Visually Impaired Athletes
Age (years), mean ± SD (range)	25.4 ± 9.1 (14–40)
Sex, *n* (%)	
Male	5 (62.5)
Female	3 (37.5)
Blindness, *n* (%)	
Congenital	6 (75.0)
Acquired	2 (25.0)
Visual disability level, *n* (%)	
Blind	3 (37.5)
Severely sight-impaired	3 (37.5)
Mildly sight-impaired	2 (25.0)

**Table 2:** Goniometric active range of motion scores at baseline and post-adapted athletic training protocol.

ROM Degrees	Baseline Mean ± SD (SEM)	Post-AAT Mean ± SD (SEM)	*p* *
Right shoulder			
Flexion	144.00 ± 13.56 (4.79)	159.12 ± 19.35 (6.84)	0.036
Abduction	159.50 ± 24.12 (8.52)	167.50 ± 19.08 (6.74)	0.078
External rotation	55.87 ± 14.96 (5.28)	54.00 ± 12.95 (4.57)	0.351
Left shoulder			
Flexion	154.12 ± 13.98 (4.94)	161.00 ± 17.01 (6.01)	0.128
Abduction	157.62 ± 30.25 (10.69)	168.12 ± 18.88 (6.67)	0.068
External rotation	58.37 ± 8.99 (3.17)	64.87 ± 6.87 (2.43)	0.075
Right elbow			
Flexion	137.87 ± 11.72 (4.14)	143.12 ± 10.32 (3.65)	0.172
Left elbow			
Flexion	135.75 ± 10.29 (3.63)	142.00 ± 7.32 (2.59)	0.080
Right wrist			
Flexion	66.50 ± 18.33 (6.48)	76.50 ± 14.17 (5.01)	0.042
Left wrist			
Flexion	64.00 ± 20.79 (7.35)	75.25 ± 13.59 (4.80)	0.075
Right hip			
Flexion	78.25 ± 12.60 (4.45)	89.12 ± 15.41 (5.44)	0.048
Extension	26.75 ± 5.25 (1.85)	31.87 ± 2.10 (0.74)	0.027
Left hip			
Flexion	77.37 ± 11.56 (4.08)	89.62 ± 17.75 (6.27)	0.046
Extension	24.50 ± 6.23 (2.20)	30.37 ± 2.55 (0.90)	0.041
Right knee			
Flexion	106.12 ± 18.02 (6.37)	109.37 ± 21.61 (7.64)	0.553
Left knee			
Flexion	105.25 ± 24.84 (8.78)	108.12 ± 13.87 (4.90)	0.833

Abbreviations: AAT, adapted athletic training; ROM, range of motion; SD, standard deviation of the mean; SEM, standard error of the mean. * Wilcoxon signed-rank test.

**Table 3:** Mean scores of running speed, pitching length, Cauquil-De Gunsch test, and Borg CR10 scale of visually impaired baseball athletes at baseline and post-adapted athletic training protocol.

Variables	Baseline Mean ± SD (SEM)	Post-AAT Mean ± SD (SEM)	*p* *
Home plate–first base speed (m/s)	4.41 ± 0.39 (0.13)	4.53 ± 0.60 (0.21)	0.400
Home plate–second base speed (m/s)	4.38 ± 0.54 (0.19)	4.14 ± 0.75 (0.26)	0.161
Orthostatic pitching length (m)	14.06 ± 3.43 (1.21)	15.03 ± 3.38 (1.19)	0.104
Kneeling pitching length (m)	12.04 ± 3.99 (1.41)	13.21 ± 3.43 (1.21)	0.161
Libra CDG test (%)			
Bipodalic stance	80.85 ± 10.52 (3.72)	75.11 ± 9.39 (3.31)	0.012
Right monopodalic stance	77.91 ± 10.11 (3.57)	72.67 ± 11.19 (3.95)	0.017
Left monopodalic stance	82.18 ± 5.40 (1.90)	77.57 ± 4.74 (1.67)	0.016
Borg CR10 scale	8.37 ± 1.06 (0.37)	7.25 ± 1.16 (0.41)	0.024

Abbreviations: AAT, adapted athletic training; SD, standard deviation of the mean; SEM, standard error of the mean; CDG test, Cauquil-De Gunsch test. * Wilcoxon signed-rank test.

## Data Availability

All relevant data are included within the manuscript.

## Supplementary Materials

The following supporting information can be downloaded at: https://www.mdpi.com/article/10.3390/ijerph20126166/s1, Examples of workout schedules of running and pitching training.
