# Supplementary material for: Reactive Agility and Pitching Performance Improvement in Visually Impaired Competitive Italian Baseball Players: An Innovative Training and Evaluation Proposal
Source: Int J Environ Res Public Health. 2023 Jun 18;20(12):6166. doi: 10.3390/ijerph20126166 (PMC10298231; doi:10.3390/ijerph20126166)
Supplement: Supplementary file 1 [file ijerph-20-06166-s001.zip › ijerph-2262458-supplementary.pdf]

RUNNING PERFORMANCE - SPECIFIC WORKOUT SCHEDULE

- **Methodological approach:** on field circuit training
- **Number of stations:** 4 (2 visually impaired athletes and a sighted trainer for each station)
- **Exercise time on each station:** 2 minutes (no recovery during station transitioning)
- **Total rounds:** 4

Station 1: Simulated jump rope movement following the pace provided by the clapping sighted trainer. Change jump technique (joined feet and alternate footstep) at each circuit round. Focus on feet-ground contact time and auditory input reactivity.

Station 2: Sprint training against progressive elastic resistance (7-9-12-15 Kg) induced by a latex elastic band wrapping athlete hip and back anchored to field fence. Perform it following the direction (straight ahead, 30 degrees right/left) verbally provided by the sighted trainer. Active recovery walking backward to the starting point between sprints. Increase elastic resistance at each circuit round. Focus on balance control, speed, and forward propulsion.

Station 3: Monopodalic stance on skimmy while performing contralateral lower limb knee-up. Stop action and keep monopodalic stance whenever the sighted trainer provides an agreed auditory input. Change support limb at each circuit round. Focus on core activation, balance control, movement speed and upper-lower limb coordination.

Station 4: Perform a single burpee and a subsequential forward sprint following and reaching the auditory input provided by the clapping sighted trainer. Active recovery walking back to starting point following the trainer verbal instructions. Add 1 burpee and increase auditory input distance at each circuit round. Focus on balance recovery during burpee-sprint transition, on reactive agility and auditory input location accuracy.

PITCHING PERFORMANCE - SPECIFIC WORKOUT SCHEDULE

- **Methodological approach:** on field circuit training
- **Number of stations:** 4 (2 visually impaired athletes and a sighted trainer for each station)
- **Exercise time on each station:** 2 minutes (no recovery during station transitioning)
- **Total rounds:** 4

Station 1: Simulated overhead pitching gesture grabbing the handle of a 2-3 kg resistance elastic tube back anchored to field fence and following the auditory target provided by the sighted trainer placed in front of athletes. Orthostatic position, bilateral execution changing arm at each circuit round. Focus on upper limb overhead control both during late cocking/acceleration and deceleration phase.

Station 2: BXC regular baseballs throwing in kneeling position following the auditory target provided by the sighted trainer placed behind the field backstop net. Dominant upper limb execution only. Focus on pitching aim accuracy and auditory target reaching.

Station 3: Simulated pitching gesture performed grabbing and using a rag to hit a baseball bat horizontally held right in front of athletes (at shoulder-height approximately) by the sighted trainer. Dominant upper limb execution only. Focus on accuracy and speed of gesture last phase, namely wrist extension-flexion.

Station 4: Athletes in orthostatic position holding a regular BXC baseball. Following the sighted trainer verbal instructions, perform a simulated right or left defensive diving, then quickly reach the required position (orthostatic or kneeling) and throw the ball aiming the auditory target frontally provided by the trainer. Dominant upper limb execution only. Focus on position transitioning speed, balance recovery and core stabilization.
